# How noncrossover homologs are conjoined and segregated in Drosophila male meiosis I: Stable but reversible homolog linkers require a novel Separase target protein

**DOI:** 10.1371/journal.pgen.1008997

**Published:** 2020-10-01

**Authors:** Elsie E. Adams, Qiutao He, Bruce D. McKee

**Affiliations:** 1 Department of Biochemistry, Cellular and Molecular Biology, University of Tennessee, Knoxville, Tennessee, United States of America; 2 Genome Science and Technology Program, University of Tennessee, Knoxville, Tennessee, United States of America; College de France CNRS, FRANCE

## Background: Segregation of chiasmate versus achiasmate bivalents

**Fig 1:**
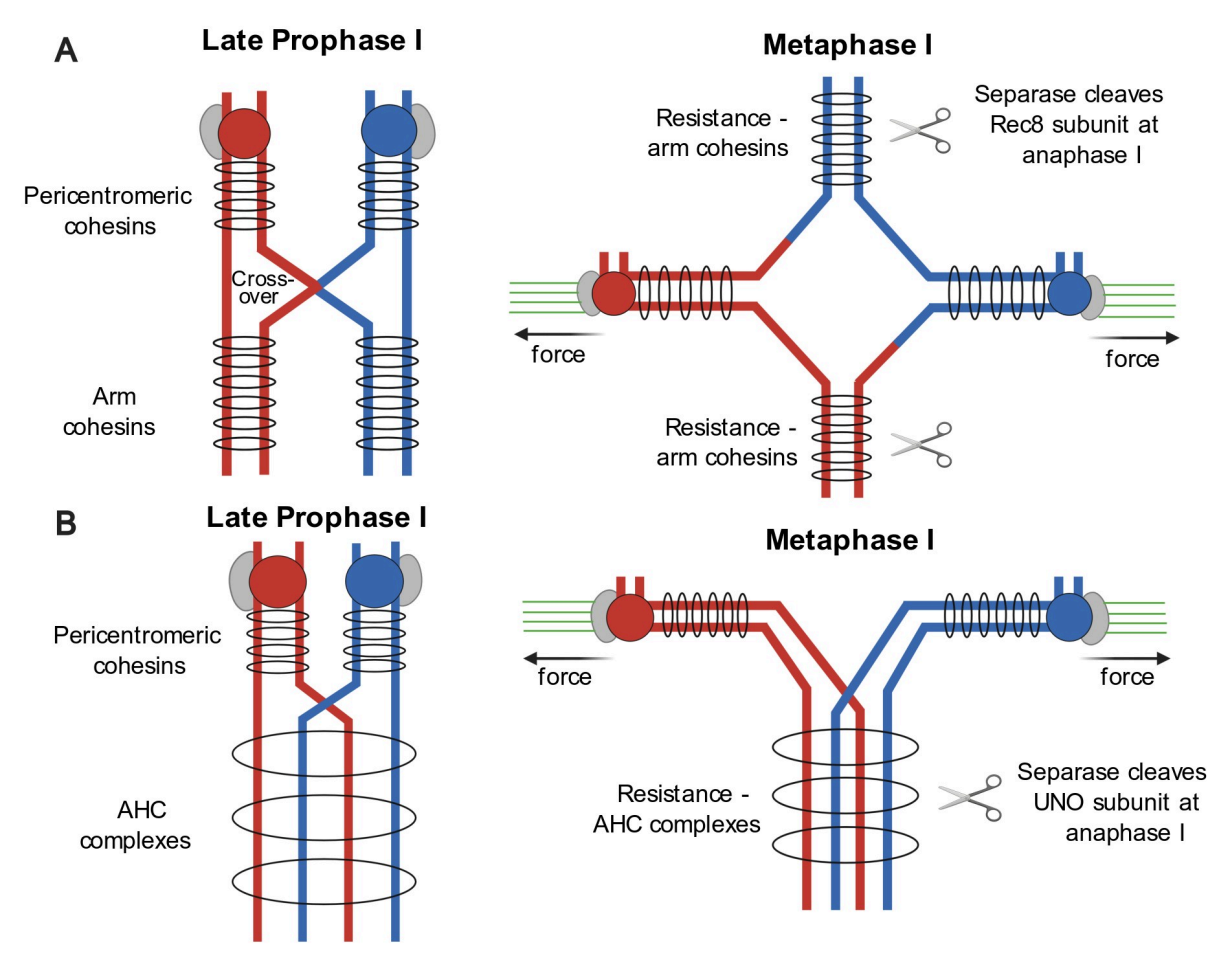
Two distinct mechanisms for linking homologous chromosomes during meiosis I. (A) Chiasmata [1]. (Left) Crossovers between non-sister chromatids (blue and red lines) are completed during prophase I. Homologs remain connected by arm cohesion provided by cohesin complexes (gray rings) located distal to sites of crossovers. Other cohesins in pericentromeric regions function to maintain sister chromatid connections until anaphase II. (Right) At metaphase I, spindle fibers (green lines) connect cooriented sister kinetochores (gray ovals) of each homologous centromere (red and blue balls) to opposite spindle poles. Opposing poleward forces (black arrows) exerted on homologous centromeres are resisted by distal arm cohesins, preventing premature separation of homologs and enabling homolog pairs to biorient. At anaphase I, Separase (scissors) cleaves the alpha-kleisin subunits of arm cohesins, dissolving chiasmata and releasing homologs to segregate to opposite poles but leaving pericentromeric cohesion intact until anaphase II. (B) AHC [5]. (Left) In Drosophila male meiosis, homologs replicate and pair but do not undergo crossing over. AHC complexes (depicted as large gray rings for illustration purposes only) composed at least of the SNM, MNM, and UNO proteins, maintain stable connections between achiasmate homologs by an unknown mechanism. (Right) At metaphase I, opposing poleward forces exerted by spindle fibers emanating from opposite poles on homologous centromeres are resisted by AHC complexes, preventing premature separation of homologs and enabling homolog pairs to biorient. At anaphase I, Separase cleaves the UNO subunits of AHC complexes, dissolving conjunction and releasing homologs to segregate to opposite poles but leaving pericentromeric cohesion intact until anaphase II [3]. AHC, alternative homolog conjunction; MNM, *mod(mdg4) in meiosis*; SNM, *stromalin in meiosis*; UNO, *univalents only*.

Segregation of homologous chromosomes is the defining event of meiosis and is central to sexual reproduction. To ensure regular segregation, homologs must establish strong but reversible physical links capable of providing the resistance to poleward spindle forces needed for stable biorientation of homologous centromeres. What form do such linkages take? In the most widespread mechanism, the linkers are chiasmata, in which crossovers between homologous chromatids are anchored by sister chromatid cohesion distal to the crossover (see Fig 1A for details) [1,2]. The anchoring cohesin complexes are similar in structure and function to the pericentromeric cohesins that enable mitotic sister chromatids to biorient and segregate properly. Cohesins are ring-shaped complexes that embrace pairs of sister chromatids at their inception and keep them together throughout the cell cycle until anaphase, when cleavage of the alpha-kleisin subunit (usually Rad21/SCC1 (Sister chromatid cohesion protein 1) or its meiosis-specific paralog Rec8) by Separase breaks the cohesin ring and releases the entrapped chromatids [1,2]. When activated at anaphase I, Separase specifically cleaves distal cohesins, thereby dissolving the chiasmata and releasing homologs (Fig 1A) [1]. Thus, chiasmata can be viewed as devices that repurpose sister chromatid cohesins as homolog linkers. This raises an important question—are there alternative mechanisms that link homologs directly, and if so, how do they work?

This question has led some researchers, including the authors of a paper in the current issue of *PLOS Genetics* [3], to investigate a variant form of meiosis in which crossing over and therefore chiasmata are absent [4]. In the best-known example of “achiasmate meiosis” in Drosophila males, a process known as “conjunction” or “alternative homolog conjunction (AHC)” yields stable bivalents that undergo nearly error-free biorientation and segregation during meiosis I (Fig 1B) [5]. Although conjunction is not yet understood in mechanistic detail, it depends on 3 previously identified genes: *stromalin in meiosis (snm)*, *mod(mdg4) in meiosis (mnm)*, and *teflon (tef)* [6,7]. Null mutations in *snm* or *mnm* do not affect homolog pairing in early prophase I but disrupt all 4 bivalents beginning in late prophase I [7,8]. Up to 8 univalents are present at prometaphase I and metaphase I, and the univalents assort randomly at anaphase I [7]. Mutations in *tef* cause the same phenotypes but affect only the 3 autosomal bivalents [6]. SNM and MNM proteins are present throughout meiosis I and colocalize, in a codependent manner, to faint foci of variable size and number on the autosomal bivalents and to a single large, dense focus associated with the pairing domain (the rRNA genes) of the X–Y bivalent [7,9]. These foci persist until the metaphase I–anaphase I transition, when their disappearance coincides with the onset of homolog segregation [7]. Unexpectedly, both the removal of SNM and MNM and the segregation of homologs at anaphase I were recently found to depend on Separase [10].

## UNO unites conjunction and Separase

Although existing data indicated roles of SNM and MNM in homolog linkage, both the mechanism of conjunction and the means by which it is removed were unclear. With the goal of identifying additional conjunction proteins, the authors of the current paper conducted a proteomic screen for testis proteins that interacted with either SNM-enhanced green fluorescent protein (EGFP) or MNM-EGFP [3]. Among the 123 proteins that interacted strongly with both baits but not with an EGFP control was the product of *CG8712* (renamed *univalents only* (*uno*)), an uncharacterized gene expressed mainly in testes. UNO lacks conserved domains, but extensive genetic and cytological characterization revealed it to be a new conjunction protein remarkably similar in function to MNM and SNM [3,7]. *uno* mutations were found to cause the same primary phenotype as *snm* and *mnm* mutations, premature dissolution of all 4 bivalents and random assortment of the resulting univalents. The spatial–temporal localization pattern of UNO protein recapitulated that of SNM and MNM, and the chromosomal foci formed by all 3 proteins were observed to be fully codependent (and dependent on *tef* on autosomes). These features strongly suggest that SNM, MNM, and UNO form a complex, but, until now, there has been no direct evidence. In support of this idea, the native MNM and SNM proteins were among the strongest interactors with both baits. Moreover, TEF copurified with MNM-EGFP, consistent with it being a component of autosomal AHC complexes [3].

The finding that Separase is required for release of conjunction at anaphase I [10] was surprising because the only known substrate of Drosophila Separase (aside from the Separase regulator Three Rows) was the mitotic alpha-kleisin Verthandi/Rad21, which appears to have only a very limited role in meiosis [11–13]. This suggested that one of the conjunction proteins might be a novel Separase substrate. Pursuing this possibility, the authors identified a conserved match for the consensus Separase cleavage site (E/DxxR) in UNO and went on to demonstrate that UNO is cleaved by Drosophila Separase and that the cleavage of UNO at anaphase I is required for homolog segregation (Fig 1B). This important finding adds to the very short list of proven Separase substrates [1,3] and clarifies how conjunction is removed [3]. The authors considered but rejected the possibility that UNO is an alpha-kleisin. Like SNM and MNM, UNO is not required for meiotic cohesion. Moreover, UNO lacks both the sequence homology and secondary structure elements present in the N- and C-termini of alpha-kleisins that are critical for their interactions with other cohesin subunits [2,3].

## Unanswered questions

As a result of recent progress exemplified by the featured paper [3], several key aspects of conjunction have come into sharper focus. (1) The conjunction proteins SNM, MNM, and UNO interact as subunits of a highly interdependent AHC complex. Detailed characterization of the composition and structure of this complex will likely yield key insights into the mechanism of conjunction and should be a top priority. (2) The only known function of the AHC complex is to maintain connections between achiasmate homologs from mid-late prophase I until anaphase I in male meiosis. Yet this is no simple function. A special challenge will be to understand how the same complex (or different complexes involving the same proteins) can accomplish this task both before chromosome condensation, when the homologs are not tightly paired and are spread throughout the approximately 200 um^3^ volume of an amorphous “chromosome territory” and after chromosome condensation when they are compacted into dense “blobs” and subjected to spindle forces [14,15]. (3) Although UNO is a Separase target, it is not an alpha-kleisin. Thus, unlike chiasmata, the AHC complex is not a repurposed cohesin, and conjunction does not depend on sister chromatid cohesion. Notably, however, this does not rule out the possibility that the AHC complex forms a cohesin-like ring that in some way corrals both homologous and sister chromatids (Fig 1B). Building on recent progress, it should soon be possible to implement tests of this and other models of conjunction.
